# gammaBOriS: Identification and Taxonomic Classification of Origins of Replication in Gammaproteobacteria using Motif-based Machine Learning

**DOI:** 10.1038/s41598-020-63424-7

**Published:** 2020-04-21

**Authors:** Theodor Sperlea, Lea Muth, Roman Martin, Christoph Weigel, Torsten Waldminghaus, Dominik Heider

**Affiliations:** 1 Faculty of Mathematics and Computer Science, University of Marburg, Hans-Meerwein-Str. 6, D-35032 Marburg, Lahn Germany; 2 Institute of Biotechnology, Faculty III, Technische Universität Berlin (TUB), Straße des 17. Juni 135, D-10623 Berlin, Germany; 3 Chromosome Biology Group, LOEWE Center for Synthetic Microbiology (SYNMIKRO), Philipps-Universität Marburg, D-35043 Marburg, Lahn Germany

**Keywords:** Machine learning, Genome informatics, Comparative genomics, Data mining

## Abstract

The biology of bacterial cells is, in general, based on information encoded on circular chromosomes. Regulation of chromosome replication is an essential process that mostly takes place at the origin of replication (*oriC*), a locus unique per chromosome. Identification of high numbers of *oriC* is a prerequisite for systematic studies that could lead to insights into *oriC* functioning as well as the identification of novel drug targets for antibiotic development. Current methods for identifying *oriC* sequences rely on chromosome-wide nucleotide disparities and are therefore limited to fully sequenced genomes, leaving a large number of genomic fragments unstudied. Here, we present gammaBOriS (Gammaproteobacterial *oriC* Searcher), which identifies *oriC* sequences on gammaproteobacterial chromosomal fragments. It does so by employing motif-based machine learning methods. Using gammaBOriS, we created BOriS DB, which currently contains 25,827 gammaproteobacterial *oriC* sequences from 1,217 species, thus making it the largest available database for *oriC* sequences to date. Furthermore, we present gammaBOriTax, a machine-learning based approach for taxonomic classification of *oriC* sequences, which was trained on the sequences in BOriS DB. Finally, we extracted the motifs relevant for identification and classification decisions of the models. Our results suggest that machine learning sequence classification approaches can offer great support in functional motif identification.

## Introduction

Before every cell division, bacteria need to duplicate their genetic material to ensure that this information can faithfully be passed on to both daughter cells. This essential process, called DNA replication, initiates in a highly regulated manner at chromosomal sites called *oriC* and is coordinated with many other cellular processes^1,2^. With notable exceptions as e.G. Vibrionales, usually, bacteria contain one or multiple copies of a single chromosome, which carries a single *oriC* sequence^3,4^.

Since many different proteins need to bind to and act upon *oriC* for initiation to occur, *oriC* contains many protein binding sites and DNA motifs^5,6^. While there is a high level of variation between *oriC* sequences of different organisms, there are also nearly universally occurring DNA motifs in *oriC* sequences^7–9^. Central among these are 9 bp short DNA motifs called DnaA boxes, which act as binding sites for the initiator protein DnaA, and exhibit differing protein binding characteristics depending on the exact sequence. Starting from these motifs, DnaA polymerizes and spreads across multiple DnaA boxes and DnaA trio motifs^10^, which then, in interplay with the protein IHF^11^, leads to double helix unwinding at a closely positioned AT-rich region called DNA unwinding element (DUE) so that the replication machinery can be loaded onto the DNA^12,13^. As *oriC* contains binding sites for proteins that relay information on the status of the cell, it can be considered as a biological information compiler and processor^14,15^. Taken together, these properties make *oriC* sequences an outstanding object for the study of DNA motifs.

All currently available computational methods for the identification of *oriC* sequences in bacterial chromosomes rely on nucleotide disparities on the leading and lagging strand of the DNA double helix^16–20^. As replication usually extends from *oriC* bidirectionally, it is one of two chromosomal sites where the leading and lagging strand switch places. The most frequently used disparity, the GC skew, usually assumes a V- or inverted V-shape with its minimum or maximum, respectively, indicating the presence of *oriC*^21,22^. However, due to natural variation, the shape of the skew can only be reliably asserted when analyzing whole chromosomal sequences or large fragments thereof. Combining the GC skew with the location of DnaA boxes, Ori-Finder^23^ was successfully used to identify a wide range of bacterial *oriC* seqences and, subsequently, create the current state-of-the-art *oriC* database DoriC^24,25^.

Deep neural networks (DNNs) have been employed for tasks similar to the identification of *oriC* sequences^26–32^. However, these methods are notorious for needing big amounts of data and computing power. Support vector machines (SVMs) that perform classification based on *k*-mer (i.e., *n*-gram) counts represent a less data-intensive alternative and have even been shown to outperform DNNs for smaller datasets^33,34^. Some *k*-mer-SVMs use models of DNA models that allow mismatches or gaps while performing *k*-mer counting, taking into account the effect of natural variation^35–37^. Furthermore, most of these machine learning models can produce a list of features important for the classification task, which is, in this case, a list of most relevant motifs.

In the current study, we present a machine-learning based approach for the study of bacterial *oriC* sequences in four parts, exemplified on Gammaproteobacteria. This class of organisms contains many model organisms (e.g., *Escherichia coli*, *Vibrio cholerae*, and *Pseudomonas putida*), and causative agents for serious illnesses (such as cholera, plague, and enteritis), which makes this taxon a highly relevant study object. First, we present gammaBOriS (Gammaproteobacterial *oriC* Searcher), a tool that identifies *oriC* sequences in full chromosomes as well as chromosomal fragments of Gammaproteobacteria. Secondly, using publicly available Gammaproteobacterial chromosomal fragments as input for gammaBOriS, we gathered the largest dataset of bacterial *oriC* sequences available to date, BOriS DB. Based on this, we thirdly trained a set of machine learning models to classify these sequences according to their respective order, family, and genus. Finally, we present a list of motifs that were important for the identification and classification and show that the machine learning models presented here were able to learn biologically relevant information from the DNA sequences presented to them.

## Results

### Implementation of gammaBOriS

gammaBOriS is implemented in R and requires a Linux operating system. The front end of the website is written in jQuery in order to make gammaBOriS accessible without specific software requirements. As input file, gammaBOriS takes a fasta-formatted file containing one or more DNA sequences of any length and returns two fasta-formatted text files: One contains fragments gammaBOriS identified as *oriC* and the other contains DNA fragments for which the classifier abstained from a decision (see Methods).

**Figure 1:**
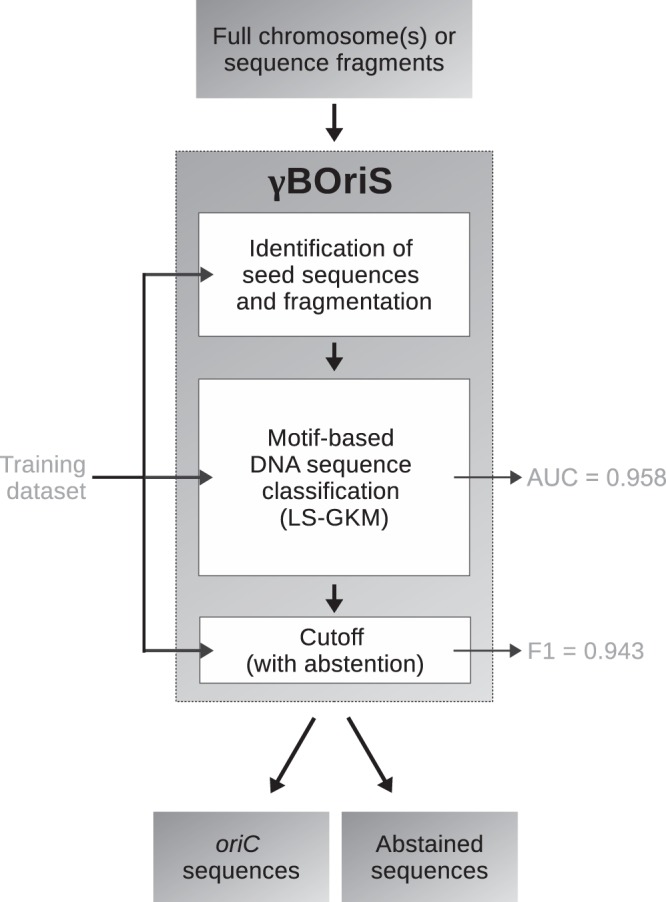
Schematic representation of the structure of gammaBOriS. Evaluation metrics on the right side of the diagram represent performance on the validation dataset.

gammaBOriS is composed of three modules that were adjusted for and trained on a training set of Gammaproteobacterial *oriC* sequences (Fig. 1). The core module consists of a *k*-mer-SVM, whose parameters were chosen to maximize the AUC of discrimination between *oriC* and non-*oriC* sequences for a balanced test dataset (for details see Methods, Fig. 1). To this end, we trained a total of 12,877 LS-GKM and spectrum kernel SVMs^33,35,36^ with varying parameters and sequence fragment sizes. We chose LS-GKM and spectrum kernel SVMs since these models can use DNA input natively, and model variations in motifs while retaining a fast runtime. The highest performance (an AUC of 0.958 on the validation dataset) was achieved with a LS-GKM model trained with 1250 bp fragments as input, a word length of 10 bp with 6 informative columns, and at most 4 mismatches (see Supplementary Figure 1, Supplementary Table 1).

To turn this sequence classifier into a sequence identifier, the first module of gammaBOriS splits the input sequence into a manageable number of candidate fragments by picking only fragments centered around an occurrence of a so-called seed sequence. A list of seed sequences was created by extracting the central 9bp sequences from each sequence in the *oriC* training dataset. This choice of seed sequences was validated by showing that all *oriC* sequences in the test dataset are centered around one of the seed sequences.

**Figure 2:**
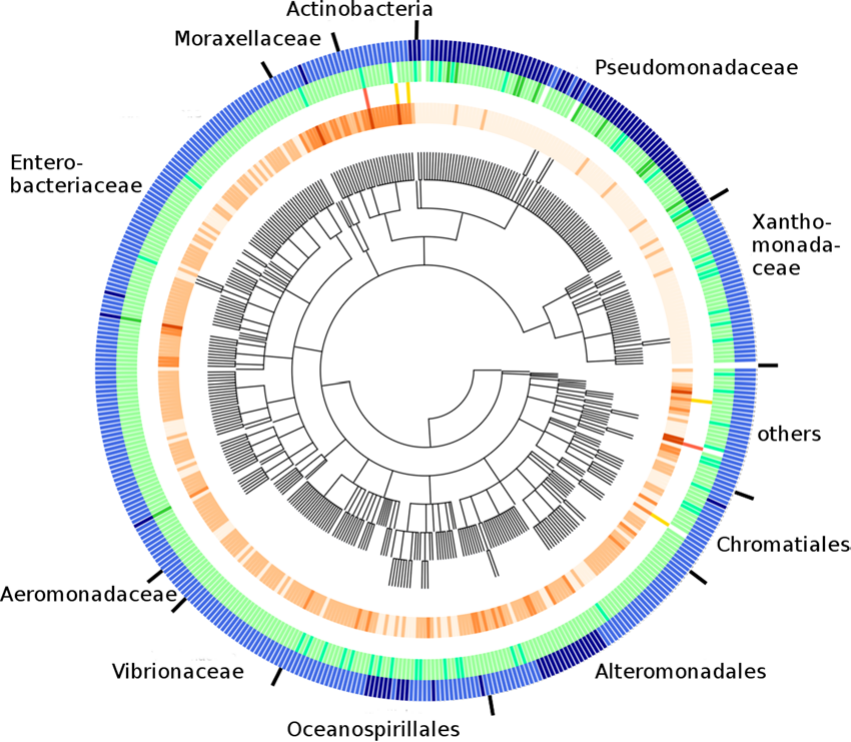
Taxonomic distribution and gammaBOriS prediction results for *oriC* sequences present in the training and test dataset (see main text for more details). Color codification, from outer to inner ring, separated by semicolons, with numbers of organisms in each category in parentheses: dark blue signifies chromosomes that contain two (105), light blue those that contain a single *oriC* sequence (355); lime green indicates that two (13) sequences were correctly identified, pale green that one (392) was correctly identified, spring green indicates that a sequence was identified that overlaps with the correct sequence (48), white indicates incorrect identification (7); red, yellow, and white indicate that two (2), one (4), and zero (454) sequences were misidentified as *oriC* sequence (false-positive), respectively; darker color indicates a higher number of candidate fragments that fall between the two cutoffs, so that gammaBOriS abstained from classification for these (min.: 1, max.: 2463, mean: 129.38).

The third module of gammaBOriS assigns a class label to every fragment based on the classification value obtained for this sequence in the second module. As, for one input sequence, the number of candidate sequences is expected to be much higher than the number of correct *oriC* sequences, this can be viewed as a highly imbalanced classification problem. To mitigate high numbers of false-positive classifications, we make use of the concept of classification with abstaining^38^. To this end, two cutoff values are employed; below the lower cutoff, fragments are labeled “negative” and discarded, above the upper cutoff, fragments are labeled “positive”, and between the cutoffs, the classifier abstains from labeling the fragments. In the choice of cutoffs, we maximized the value of the F1 metric and minimized the number of correct *oriC* sequences for which this module abstained from classification, leading to a Pareto-optimal state. We found that normalizing the classification values of the fragments extracted from one sequence to a range between [0, 1] and employing cutoffs of 0.99 and 0.41 lead to the best result on the test dataset (F1 of 0.943 with 0.7% of correct *oriC*s in the abstained space; Fig. 2, see Supplementary Figure 2). This cutoff set was also chosen to minimize the possibility of false negative classification, taking into account a slightly higher number of false-positive classifications, as false-positive sequences can still be filtered out based on domain knowledge while false negative classifications usually cannot be reverted. Similarly, sequences for which gammaBOriS abstains from classification are also returned to the user, so that other methods as well as domain knowledge can be used to reach a decision on whether a fragment contains an *oriC* sequence or not.

### Construction of BOriS DB

We then applied gammaBOriS to all chromosomes and chromosomal fragments present in the RefSeq database^39^ (restricted to sequences with the release type “Major”) as well as the genomes in the Uncultivated Bacteria and Archaea (UBA) dataset^40^. Both datasets contain a high number of incompletely sequenced chromosomes and chromosomal fragments, and therefore cannot be analyzed using previous *oriC* identification methods. After discarding sequences present in both databases, we retained 25,827 *oriC* sequences from 1,217 different gammaproteobacterial species, most of which were not identified before. These sequences constitute the first version of BOriS DB.

Since only very few *oriC* sequences are experimentally confirmed for Gammaproteobacteria, and there is no established *oriC* benchmark dataset, a direct, computational comparison of *oriC* identification tools is infeasible. In theory, it is possible to assess whether a DNA sequence functions as *oriC* using wet lab experiments, however, especially for non-model organisms, these are prohibitively costly and error-prone. Instead, we compared a subset of BOriS DB to the current state-of-the-art *oriC* database, DoriC^24,25^ (for details, see supporting information). In 67, 90% of the chromosomes, both databases contain the same *oriC* sequence. In many of the cases of disagreement, the sequences identified BOriS DB show a slightly higher degree of consistency when compared to closely related sequences (see Supplementary Figures 3 and 4), although this result is hard to interpret due to a lack of experimentally confirmed *oriC* sequences and an established *oriC* benchmark dataset.

We conclude that BOriS DB, while only containing sequences from Gammaproteobacteria, is the largest database of *oriC* sequences to date and is at least as exact as DoriC.

### Taxonomic Classification of *oriC* sequences

To make use of the information gathered in BOriS DB, we constructed a machine learning model that classifies gammaproteobacterial *oriC* sequences taxonomically at the levels of order, family, and genus. To this end, we employed nine different machine learning models, which include LS-GKM and Random Forests (RFs)^36,41^. For the latter, sequences were encoded using one-hot encoding, *k*-mer counting with 1 ≤ *k* ≤ 6, and a word2vec model^42^. Word2vec aims at deriving semantic information from the syntactic position of words (here: *k*-mers) and has shown great promise for natural language. Since taxonomic classification is an imbalanced multi-class classification problem, we chose macroAUPR as evaluation metric, which is calculated by averaging over the AUPR values of all classes of a taxonomic level.

**Figure 3:**
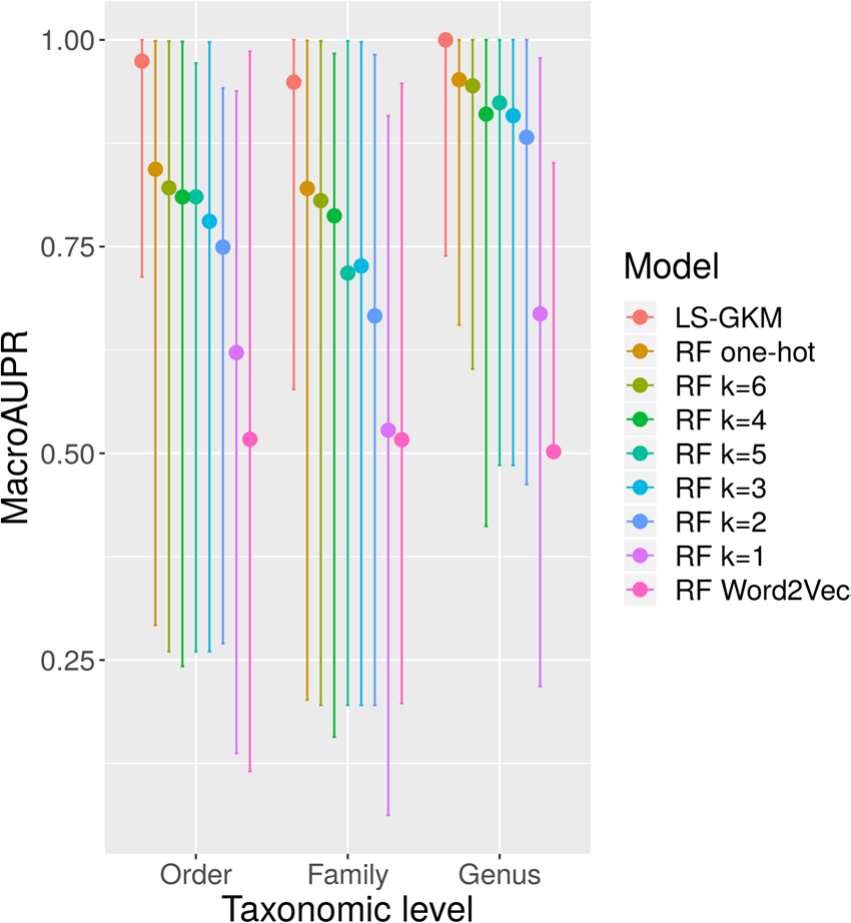
Evaluation of different models for taxonomic classification of *oriC* sequences present in BOriS DB. MacroAUPR designates the average of the area under the precision-recall curve, a common metric for imbalanced multi-class classification tasks. RF stands for Random Forest. Error bars represent the standard deviation of the AUPR values achieved for the different taxa.

The results show that, consistent across the different taxonomic levels, LS-GKM outperforms the other methods and achieves macroAUPR values of ≥0.9. RFs trained on word2vec-derived encodings, on the other hand, show the worst performance (Fig. 3). This might be because, in contrast to Random Forests, neural network-based machine learning approaches require a high amount of training data. Furthermore, there are structural differences between DNA sequences and natural languages, for which word2vec was originally designed. The fact that all models perform better at the level of order than at the level of family or genus suggest that the information present in *oriC* sequences is more specific at the genus level than at the others. It is also noteworthy that RFs trained on one-hot encoded sequences outperformed those trained on *k*-mer counting encodings, as this suggests that the position of certain motifs in the sequence is important for taxonomic classification.

Since LS-GKM models also outperformed RF models when evaluated in regards to single taxa (see Supplementary Figures 5–7), we decided to use the former for gammaBOriTax, a tool for taxonomic classification of gammaproteobacterial *oriC* sequences. gammaBOriTax is available at boris.heiderlab.de both as a stand-alone tool as well as for automatic post-processing of the output of gammaBOriS.

### Motif extraction

Like many other machine learning approaches, LS-GKM allows for the extraction of feature importance values that describe the relevance of motifs for the classification decision. By extracting the feature importance values from the LS-GKM model at the core of gammaBOriS, and discarding all motifs with a negative importance value, we obtained 74 motifs (see Supplementary Table 2 for a full list). Grouping these motifs by sequence similarity and common substrings results in four motif classes:

(i) 28 motifs can be summarized to AAAGATCTTT. This sequence contains the motif GATC, which has many different functions in Gammaproteobacteria^43–45^; also, substrings of this consensus sequence are present in most of the sequences in BOriS DB,
(ii) 27 motifs belong to a group of AT-rich motifs that form the consensus TAATAATAA (or, if allowing for ambiguous bases, ATAWWHATA), which constitutes the DnaA trio motif^10^,
(iii) 15 motifs belong to a group of motifs that is reverse-complementary to those in class (ii),
(iv) 8 motifs can be summarized to the consensus sequence TTCTGTGGATA, which is the sequence of a high affinity DnaA box (R1 and R4 in the *oriC* sequence of *E. coli*)^46,47^.

As these four motifs cover most of the known functional classes of motifs in *oriC* sequences, this result shows that the LS-GKM models can learn biologically relevant motifs during training, even if the models has had no access to knowledge about the biological function of the motifs.

We then employed the motif extraction process to the LS-GKM models trained for taxonomic classification. However, only for 13 models we obtained any positively valued *k*-mers (see Supporting Table 3 for a full list). We noticed that many of these displayed large overlaps (i.e., common substrings without any mutations at the end of one and the beginning of another *k*-mers) with other *k*-mers extracted from the same taxon and therefore decided to assemble them according to these overlaps. The resulting motifs are presented in Table 1, together with functional annotations derived by comparison to motifs of known function present in *oriC*. The fact that these motifs represent many of the known functional components of gammaproteobacterial *oriC* sequences further supports the finding that LS-GKM models can learn biologically relevant information from sequence alone.

**Table 1:** Consensus sequences of important motifs extracted from LS-GKM models trained for taxonomic classification.

Taxonomic level	Taxon	Taxonomically Relevant Motifs		Annotation
Order	Alteromonadales	**T**AT**T**AC**T**GT**T**AT**T**	AATAACAGTAATA	DnaA trio
		AGATCTTAAGATCT		DUE
	Pseudomonadales	**TATCCACA**GAA	TTC**TGTGGATA**	DnaA box (R1)
		plus: AT-rich, ungroupable *k*-mers		DUE
	Vibrionales	AA**ATGATCA**A	T**TGATCAT**TT	RctB binding site
	Xanthomonadales	GTGGT**GGTGGTRR**TGGT	ACCA**YYACCACC**ACCAC	DnaA box
Family	Enterobacteriaceae	TAAGAGATCA	TGATCTCTTA	DnaA trio
	Halomonadaceae	ACAGAACTTC	GAAGTTCTGT	DUE
	Pseudomonadaceae	TATA**A**AGCTT**A**WTA	TAWAAAGCTTATA	DnaA trio
		**TATCCACA**GA	TC**TGTGGATA**	DnaA box (R1, R4 or p7/8)
	Vibrionaceae	AA**ATGATCA**A	T**TGATCAT**TT	RctB binding site, DnaA Box (R1)
	Xanthomonadaceae	GTGGT**GGTGGTGAT**	**ATCACCACC**ACCA	DnaA box (R1)
Genus		**AA**GC**TGTGGA**	**TCCACA**GC**TT**	DnaA box (R2, R4)
	Acinetobacter	TAAATTTAAATTTA		DnaA trio
	Escherichia	CAAGGATCCAGCTTTTAA**GAT**	**ATC**TTAAAAGCTGGATCCTTG	IHF binding site
	Pseudomonas	TC**TATCCACA**GAA	TTC**TGTGGATA**GA	DnaA box (R1)
	Shigella	CAAGGATCCGATTTTTAA**GAT**	**ATC**TTAAAAATCGGATCCTTG	IHF binding site
		CGCACTACCCTGTGGA	TCCACAGGGTAGTGCG	DnaA trio

Reverse complementary motif pairs are displayed in the same row. Annotation of the motifs is based on substrings highlighted in bold, except for motifs annotated with DUE, which were annotated based on their location in the downstream unwinding element of *oriC* sequences from the respective taxon. Due to the fact that LS-GKM models ambiguous bases, the consensus sequences presented here are not necessarily present in *oriC* sequences with this exact sequence. The motifs annotated as RctB binding site conform to the consensus sequence NNNNNNWTGATCATKSWT. DnaA box annotation appendices were adapted from Grimwade *et al*.^71^.

It is noteworthy that the tetramer GATC is only present in motifs extracted from taxa that contain a Dam methyltransferase gene^48,49^. Dam methylates GATC motifs and is implicated in many processes that regulate replication initiation and DNA repair^43^. Surprisingly, for Vibrionales and Vibrionaceae, we obtained a motif that closely resembles the binding site consensus sequence for the initiator protein for the secondary chromosomes of these organisms, RctB^50^. The presence of a RctB binding site in *oriC* from Vibrionaceae has not been described before. Albeit the deviations in sequence might lead to a lower binding affinity, this motif might play a part in the coordination of the replication initiation between the two chromosomes^51,52^. Taken together, the results presented in this section show that, generally, LS-GKM models can learn biologically significant patterns and might be used to identify novel, functionally important motifs.

Because we were able to obtain important *k*-mers for only a few taxa, we also extracted the 20 highest-valued motifs for each taxon (for a full list see Supplementary Table 4). In these motifs, we observe a significantly higher amount of GATC motifs in taxa that do contain a Dam gene compared to those that do not (see Supplementary Figure 8). As there are no reliable databases for DNA binding sites of proteins involved in DNA replication initiation (i.e. mostly non-transcription regulatorory proteins), functional annotation of these motifs is infeasible. However, based on the results presented above, we expect that many of the motifs discovered here are specific for the respective taxon and associated with proteins essential for replication initiation.

## Discussion

The application of methods from the field of machine learning to biology hold great promise, especially for the classification and identification of DNA sequences and the effect of variants in them^53–55^. However, only few studies have used machine learning approaches to illuminate the biology of prokaryotes. E. g., while machine learning methods have already been employed for the identification of origins of replication in yeast^56^, *oriC* identification in bacterial chromosomes is based on chromosome-wide nucleotide disparities such as the GC-skew^57^. The latter, however, cannot be used for the huge number of fragmentarily sequenced genomes currently present in public databases. Furthermore, methods developed for eukaryotic chromosomes cannot be applied to bacterial chromosomes as the composition of these sequences and the processes in replication initiation are very different^58^.

In this study, we present a set of machine learning-based tools (available at http://boris.heiderlab.de) for the analysis of gammaproteobacterial chromosomes and, more specifically, the *oriC* sequences therein. We chose to focus on one class of organisms because *oriC* sequences from different taxonomic classes exhibit a high level of variance^7,59^. Moreover, as most secondary chromosomes use particular initiation proteins for replication initiation^52,60^, and as they are rather rare^3^, we also excluded these from the scope of this study and focused on primary chromosomes.

Firstly, we introduce gammaBOriS, which is able to identify *oriC* sequences on fragmentary as well as full chromosomes of Gammaproteobacteria using a motif-based machine learning method. The general approach of gammaBOriS can easily be adapted for other groups of organisms if trained on their *oriC* sequences. Suitable datasets, however, are currently not easily available in the necessary amount and quality (e.g., equal-sized, centered, and co-oriented). The method used to create an initial *oriC* dataset in this study requires manual decision-making and is thus not scaleable, but also ensures that the weight of implicit assumptions can be balanced and adjusted for every case. The assumptions of the method are that (I) *oriC* is intergenic, (II) close to the global GC skew minimum, and (III) defined by the DUE, as well as (IV) the presence of DnaA boxes. We consider this method highly accurate, which is supported by the fact that some *oriC* sequences identified with it have been confirmed experimentally^9,61,62^. Being trained on this dataset, gammaBOriS can be seen as scalable automation of the semi-automatic method, which makes it possible to use it for the analysis of large-scale metagenomic datasets^63,64^.

By applying gammaBOriS on fragments deposited in public sequence databases, we created BOriS DB, the largest database of *oriC* sequences to date. BOriS DB currently contains 25,827 sequences from 1,217 species from the class of Gammaproteobacteria, most of which were not identified yet. We exemplify the use of BOriS DB by training machine learning methods to perform taxonomic classifications of the sequences in it. Based on the best performing classifiers, we created gammaBOriTax (Fig. 3). Furthermore, we show that it is possible to extract sets of biologically relevant motifs from the models used in gammaBOriTax. While many of the motifs identified this way have a known function, there are also motifs that we cannot annotate functionally yet (Table 1). Due to their statistical properties, models employed in gammaBOriTax will only identify motifs as relevant for classification that are both specific for a given taxon and evolutionarily conserved in this taxon. Therefore, the proteins that bind to and interact with these motifs ideal potential targets for novel antibiotics^65^.

The motifs identified as relevant by gammaBOriS and gammaBOriTax are representative of all the motif classes known to be functionally important for *oriC* of Gammaproteobacteria, including the only recently identified DnaA trio motifs^10^. Furthermore, gammaBOriTax identified a motif that resembles the RctB consensus sequence and might act as a low-affinity binding site, thus playing a role in coordination between the primary and secondary chromosome in Vibrionales. Based on this, we suspect that the motifs annotated as DUE in Table 1 might not simply be AT-rich sequences but functionally important protein binding sites. While further experiments are needed to confirm these hypotheses, the results presented here show that it is possible to derive sets of biologically relevant motifs from machine learning methods trained without explicit domain knowledge.

## Methods

### Data curation and creation

A basic “ground truth” *oriC* dataset was compiled using a semi-automated method described in^9,61,62^. A given chromosome is first split into 2.5 kb fragments that are centered around intergenic regions and then, for those fragments close to the minimum of the chromosome’s cumulative GC-skew, their respective probability of unwinding is calculated using WebSIDD^66^. Default values (37 °C, 0.1 M salt, circular DNA, copolymeric) were chosen for the predictions, and negative superhelicity values were tested in the range of *σ*. In the following, the sequences identified this way are regarded as “positive” examples, i.e., sequences that do contain an *oriC* sequence.

A dataset of seed sequences was created by extracting the central 9 bp from *oriC* sequences in the ground truth dataset. Negative sequences, i.e., DNA sequences that do not contain an *oriC* sequence, were collected by picking sequences from each chromosome present in the positive dataset. These sequences were chosen to have (I) the same size as and (II) the same seed sequence as the positive sequence from the respective chromosome. To be able to identify the optimal fragment length for classification, the length of the sequences in the datasets were varied from 150 to 1500 bp in steps of 50 bp. Seed sequence and sequence datasets with a fragment length of 1250 bp are available at Figshare (see Additional Information).

For cutoff selection, a highly imbalanced sequence dataset was created by extracting all fragments of a given length around each of the seed sequences from each of the chromosomes present in the positive dataset. Both the balanced “ground truth” as well as the imbalanced datasets were split into training and test datasets using a 70–30% split, leading to 318 chromosomes in the former and 141 chromosomes in the latter. For validation of the models, we used a separate validation dataset of 100 sequences created the same way.

Chromosomes were downloaded from the NCBI RefSeq ftp server^39^. For BOriS DB, a list of RefSeq organisms was taken from ftp://ftp.ncbi.nlm.nih.gov/genomes/refseq/bacteria/assembly_ summary.txt, a list of chromosomes for the UBA genomes was taken from Supplementary 2 of Parks *et al*.^40^.

### Sequence classification using LS-GKM models

The support vector machines used as classifiers in this study derive distance matrices from a set of input sequences by counting substrings and comparing their numbers in sequence pairs directly, making these approaches fast and memory efficient. The Spectrum Kernel is based on simple *k*-mer composition differences^33^. LS-GKM and gkm-SVM models calculate differences between *k*-mers by allowing for mismatches and small differences between the *k*-mers^36,67^. The optimal parameter set for each of the models was assessed after model training on the validation dataset.

### Taxonomic classification of *oriC* sequences

Different machine learning approaches were used to classify DNA sequences taken from BOriS DB with regard to their respective taxonomic orders, families and genera. The test-train-validation data split used to train gammaBOriS was also used here. LS-GKM models were trained for binary classification for each of the taxa with more than 4 examples in BOriS DB using the same parameters that showed the best classification performance for *oriC*/non-*oriC* classification. Random Forest classifiers^41^ taken from R package caret^68^ (one-hot and *k*-mer counting encodings) and the Python package scikit-learn^69^ (word2vec encoding) were trained on one-hot encoded sequences as well as on *k*-mer counting encoded DNA sequences (for 1 ≤ *k* ≤ 6) and encodings taken from a word2vec model. Pseudo-sentences, that were used as input for Gensim-based word2vec models^70^, were generated by splitting the sequences into all possible sets of consecutive 10-mers.

### Motif extraction from LS-GKM models

The importance of motifs for LS-GKM classification decision was assessed by scoring a list of all 10-mers via the gkmpredict method provided by LS-GKM as described by the original authors of LS-GKM^36^. Motifs were deemed important if their score was larger than or equal to 0.

### Accession codes

Datasets used for model training and validation for gammaBOriS and BOriS DB v1 are available under the DOIs 10.6084/m9.figshare.8108357.v1, 10.6084/m9.figshare.10079753, and 10.6084/m9.figshare.9919145.v1.

## Supplementary information

Supplementary information.
