# Supplementary material for: gammaBOriS: Identification and Taxonomic Classification of Origins of Replication in Gammaproteobacteria using Motif-based Machine Learning
Source: Sci Rep. 2020 Apr 21;10:6727. doi: 10.1038/s41598-020-63424-7 (PMC7174414; doi:10.1038/s41598-020-63424-7)
Supplement: Supplementary file 1 — Supplementary information. [file 41598_2020_63424_MOESM1_ESM.pdf]

gammaBOriS: Identification and Taxonomic Classification of Origins of Replication in Gammaproteobacteria using Motif-based Machine Learning
Supplementary Information

Theodor Sperlea [1], Lea Muth [1], Roman Martin [1], Christoph Weigel [2], Torsten Waldminghaus [3] and Dominik Heider [1]

1. Faculty of Mathematics and Computer Science,
Philipps-Universität Marburg,
D-35043 Marburg, Germany
2. Department of Life Science Engineering,
Fachbereich 2, HTW Berlin,
D-10318 Berlin, Germany
3. LOEWE Center for Synthetic Microbiology (SYNMIKRO),
Philipps-Universität Marburg,
D-35043 Marburg, Germany

1 *k*-mer SVM training and evaluation

Models from the LS-GKM package [13] and Spectrum Kernel models [6] were trained using 70% of the *oriC* sequences in the initial dataset. A total of 13000 different sets of model parameters and sequence lengths were employed and evaluated using the remaining 30% of the datasets (see table 1 for details). Almost all models achieved an AUC of larger than 0.8 (see Supporting Figure 1). Due to the number of models trained, the difference of performance between the models is not significant (as calculated using the DeLong test [5]).

Table 1: Parameters of the machine learning models used here and the ranges of their values. Ranges are presented in square brackets that contain the start and end value and step size. Values in parentheses suggest a list of possible values.

Parameter Name	Number LS-GKM	Spectrum
Fragment Length	[100, 1500, 50]	[100, 1500, 50]
Motif length	[6, 12, 1]	[6, 12, 1]
Model type	(T0, T1, T2, T3, T4, T5)	-
Informative Columns	[5, 11, 1]	-
Allowed Mismatches	[1, 4, 1]	-
Total amount of models	12877	209

The best performing models are LS-GKM models with the type T4 and T5. Furthermore, almost all of the 25 best performing models were trained using a fragment length of 1250 bp and 6 informative columns, while there is more variation in these parameters for the 250 best performing models (see Supplementary Figure 1, Supplementary Table 1). The other parameters provided by LS-GKM, i.e. the word length and number of mismatches, seem not to have a large impact on model performance as they show a high amount of variation in the best performing models (see Supplementary Table 1).

2 Implementation of BOriS, an *oriC* identification method

2.1 Turning a classifier into an identifier

In order to transform a DNA classification model into a model that is able to identify a genomic position where the classification value is maximal, we see three different possible approaches:

1) LS-GKM models are able to use DNA fragments as inputs that have different lengths from the DNA fragments they were trained on. Therefore, one could split a large-scale DNA sequence into a few very large DNA fragments, score these using a LS-GKM model, then splitting the highest scoring one into sub-fragments and scoring these, and so on, until having reached the target fragment size. However, as it is not the case that the fragment containing *oriC* will have the highest classification score independent of fragment size (i.e. size of "noise" DNA sequences), this approach is

not reliable.

2) In order to create fragments of the size that the LS-GKM model was trained on, one could use a moving window approach, starting each candidate fragment a certain amount of base pairs after the start point of the last fragment. For this approach to work efficiently, one would need to identify the highest step size for which the classifier still identifies each *oriC* sequence. Nevertheless, this approach would lead to a number of LS-GKM scoring steps that grows linear with input sequence size. Therefore, this approach is not practical for large sequences such as bacterial chromosomes.

3) In order to choose a reduced number of candidate fragments, one might employ a filtering method, as e.g. choosing only candidates around a certain sequence pattern. Reducing the number of candidates in this way leads, of course, to a lower coverage of the input sequence, which, in turn, might lead to high numbers of false negatives, as some positive sequences might be discarded in the filtering step and not identified as candidates.

Having decided to employ the third approach, we created seed sequences that were extracted from the center of the sequences in the initial dataset and then created candidate fragments of 1250 bp centered around any one of the instances of the seed sequences in the input genome sequences. The size of the candidate fragments was chosen to fit the fragment size used to train the best-performing LS-GKM model. As the seed sequences that can be extracted from the test dataset are a subset of the seed sequences extracted from the training sequences, we conclude that our choice of seed sequences will not lead to many false negatives, which is also supported by the results shown in sect. 2.1.1.

Note that the size and composition of these seed sequence list is one of the largest contributors to the runtime of gammaBOriS; therefore, a more elegant and sparse way of defining candidate sequences would shorten the runtime of LS-GKM greatly.

2.1.1 Identification of optimal cutoffs

Using a classifier to identify a DNA fragment from a larger set of candidate sequences turns the balanced classification problem the classifier was trained on into a highly unbalanced one. Therefore, cutoffs in the classifier output value need to be identified that maximize metrics suitable for imbalanced classification problems, such as the F1 score. Here, making use of the concept of classification with abstaining (creating a range of values in which the classifier abstains from assigning a classification label to candidate sequences) [1] is a possible way to avoid a high number of false negatives while also keeping the number of false positives low.

In order to identify a cutoff setting that minimizes the number of samples for which the classifier abstained while resulting in a maximal F1 score, four different classification score normalization methods were investigated for different cutoff configurations:

- no preprocessing (”raw” in Supplementary Figure 2),
- normalizing the classification scores of the fragments gathered from one input sequence to a range of [0, 1] (”norm” in Supplementary Figure 2),
- normalizing the classification scores of the fragments gathered from one input sequence by calculating z-scores (”zscore” in Supplementary Figure 2) using the formula

  $$z = \frac{(X - \mu)}{\sigma}$$

  where x is the classification score of a fragment, μ is the mean of the classification scores of the fragments of one input sequence and σ is the standard deviation of the classification scores of the fragments of one input sequence and
- normalizing the z-score normalized classification scores of the fragments extracted from one input sequence to a range of [0, 1] (”zscore norm” in Supplementary Figure 2).

Using the same train-test split as used for model training, we identified cutoff-normalization configurations that are Pareto optimal in maximizing the F1 score while minimizing the number of rejected chromosomes using the R package rPref [17]. From this set of configurations, we decided to use the one for gammaBOriS that creates no false negative results on test data (as well as the training data), while taking a higher number of false positive decisions into account. This way, researchers using gammaBOriS will be presented some wrong fragments, but no fragments that do contain *oriC* will be among the discarded sequences. Therefore, we chose a lower cutoff of 0.41 and a upper cutoff of 0.99 for data that were normalized to a range between 0 and 1, which resulted in a F1 score of 0.94 and 11 rejected *oriC* sequences on test data.

3 Comparison of gammaBOriS DB to DoriC

Due to the fact that, currently, there is no widely accepted *oriC* benchmark as well as no objective ”ground truth” dataset, it is impossible to compare methods for the

identification of *oriC* sequences. Therefore, we decided to compare DoriC (version 6.5) [7], which was created using Ori-Finder, to the results of using gammaBOriS on the same chromosomes Ori-Finder was used on to create DoriC. Ori-Finder is the current state-of-the-art method for the identification of *oriC* sequences and makes use of the Z-curve method [9, 14].

Pairs of sequences taken from the same chromosome different in the two datasets were compared by calculating the length of the longest common substring and dividing it by the length of the shorter sequence, resulting in a value of relative sequence overlap. Two sequences were considered to be identical if the relative sequence overlap was above a cutoff of 0.7, which was chosen in order to include overlapping sequences. For 330 of the chromosomes listed in DoriC we find identical *oriC* sequences, however, for 156 there is disagreement.

The internal consistency of the sequence datasets was evaluated by calculating all-vs-all sequence similarities from pairwise sequence alignments (performed using the align submodule from Biopython [4]) after making the sequences in the datasets of same length. Using multidimensional scaling and hierarchical clustering (as implemented in the Python packages scikit-learn and scipy, respectively [11, 16]), these distance matrices were visualized. A database was deemed more consistent if the degree of clustering is higher or if *oriC* sequences from closely related organisms are close on the tree.

Visualization using multidimensional scaling shows no big difference between gammaBOriS and DoriC, although the sequences identified using the former form slightly tighter clusters (Supplementary Figure 3), which indicates more consistency. This result is also supported by phylogenetic trees derived from the pairwise distance matrices (Supplementary Figure 4). To quantify this, we calculated average sequence similarities for taxonomic orders (see tab. 2).These results show that while for most orders, gammaBOriS is more consistent than DoriC (as, e.g., for Vibrionales and Xanthomonadales), the contrary is true for chromosomes from, e.g., Methylococcales and Thiotrichales. This is, most likely, due to low numbers of organisms from these groups in the initial dataset (especially for Thiotrichales and Methylococcales). Chromosomes of Pseudomonadales are known to contain two *oriC*, whose function is not fully clear yet (Are both needed for correct initiation or can initiation occur at both? Do the loci need to interact for initiation to occur?) [10, 8]. In the ground truth dataset used for training and testing of gammaBOriS, both sequences are present; however, only one of the sequences is usually identified as *oriC* by gammaBOriS (see fig. 2 in the main paper). This might reflect a difference in function of these two *oriC* sequences, but further research is necessary here.

Taken together, these results show that a comparison between DoriC and gamma-

Table 2: Mean and standard deviation of the scores of pairwise global alignments in taxonomic groups as an indication of consistency. A higher score indicates a higher degree of similarity.

Taxon	BOriS DB		DoriC	
	Mean	St.dev.	Mean	St.dev.
Aeromonadales	1032.16	146.23	977.33	158.6
Alteromonadales	912.01	352.23	1119.65	130.35
Cellvibrionales	927.5	194.55	910.75	202.47
Chromatiales	861.74	137.19	851.64	134.12
Enterobacterales	982.66	149.34	977.19	150.67
Legionellales	1005.09	211.07	983.37	203.7
Methylococcales	940.64	189.9	969.11	218.48
Oceanospirillales	823.4	145.01	821.46	137.68
Pasteurellales	947.3	105.91	920.69	106.99
Pseudomonadales	849.24	117.9	886.47	132.95
Thiotrichales	909.72	156.8	985.57	185.74
Vibrionales	1006.08	118.29	927.43	127.3
Xanthomonadales	987.11	140.4	910.18	140.55

BOriS doesn't result in a clear ranking between the two databases.

4 Taxonomic classification of *oriC* sequences

Taxonomic classification of *oriC* sequences was attempted using different machine learning models, including LS-GKM [13] and Random Forests [2] with different sequence encodings. These numerical encodings include

- In one-hot encoding of DNA sequences, each position of a sequence is represented by a vector of length four, where each position represents one of the four possible bases and assumes a value of 1 if this base is present in the sequence at this position and 0 if not. The vectors of the single bases are then concatenated.

- A k-mer counting encoding is a vector of size 4^k where each position assumes the value of the number of occurrences of a k-mer (i.e. n-gram) divided by the total number of counted k-mers. Here, we used k-mer counting with values for k of $1 \leq k \leq 6$.

- Word2vec-based encodings were first used to assign a vector to each word in a corpus of text and have surprising properties, such as representing meaning of the words [15]. The encodings are derived from the hidden layer of a shallow neural network trained to predict the surrounding words of the words in a corpus. We trained a word2vec model from the Gensim package with a hidden layer size of 100, a window of 3 and using skip-gram, on the DNA sequences in BOriS DB split into 10-mers with frame shifts by spaces. parameters

Random Forests were taken from the R-package caret [12], except for the models trained with word2vec encodings, which were taken from the Python package scikit-learn [16].

The results for taxonomic classification on different taxonomic levels (see Supplementary Figure 5, Supplementary Figure 6 and Supplementary Figure 7) show that, in most cases, LS-GKM outperforms all other models while the word2vec-based Random Forests perform worst.

References

[1] Akshay Balsubramani. The utility of abstaining in binary classification. *arXiv*, 12 2015.

[2] Leo Breiman. Random forests. *Machine Learning*, 45(1):5–32, 2001.

[3] P. Brezellec, M. Hoebeke, M.-S. Hiet, S. Pasek, and J.-L. Ferat. DomainSieve: a protein domain-based screen that led to the identification of dam-associated genes with potential link to DNA maintenance. *Bioinformatics*, 22(16):1935–1941, jun 2006.

[4] P. J. A. Cock, T. Antao, J. T. Chang, B. A. Chapman, C. J. Cox, A. Dalke, I. Friedberg, T. Hamelryck, F. Kauff, B. Wilczynski, and M. J. L. de Hoon. Biopython: freely available python tools for computational molecular biology and bioinformatics. *Bioinformatics*, 25(11):1422–1423, mar 2009.

[5] Elizabeth R. DeLong, David M. DeLong, and Daniel L. Clarke-Pearson. Comparing the areas under two or more correlated receiver operating characteristic curves: A nonparametric approach. *Biometrics*, 44(3):837, sep 1988.

[6] Abdulkadir Elmas, Xiaodong Wang, and Jacqueline M. Dresch. The folded k-spectrum kernel: A machine learning approach to detecting transcription factor binding sites with gapped nucleotide dependencies. *PLOS ONE*, 12(10):e0185570, oct 2017.

[7] F. Gao, H. Luo, and C.-T. Zhang. DoriC 5.0: an updated database of oriC regions in both bacterial and archaeal genomes. *Nucleic Acids Research*, 41(D1):D90–D93, oct 2012.

[8] Feng Gao. Bacteria may have multiple replication origins. *Front. Microbiol.*, 6, apr 2015.

[9] Feng Gao and Chun-Ting Zhang. Ori-finder: A web-based system for finding oriCs in unannotated bacterial genomes. *BMC Bioinformatics*, 9(1):79, 2008.

[10] Yong Jiang, Shiyin Yao, Donald Helinski, and Aresa Toukdarian. Functional analysis of two putative chromosomal replication origins from pseudomonas aeruginosa. *Plasmid*, 55(3):194–200, may 2006.

[11] Eric Jones, Travis Oliphant, Pearu Peterson, et al. SciPy: Open source scientific tools for Python, 2001–.

[12] Max Kuhn. Building predictive models inRUsing thecaretPackage. *Journal of Statistical Software*, 28(5), 2008.

[13] Dongwon Lee. LS-GKM: a new gkm-SVM for large-scale datasets. *Bioinformatics*, 32(14):2196–2198, mar 2016.

[14] Hao Luo, Chun-Lan Quan, Chong Peng, and Feng Gao. Recent development of ori-finder system and DoriC database for microbial replication origins. *Briefings in Bioinformatics*, jan 2018.

[15] Tomas Mikolov, Kai Chen, Greg Corrado, and Jeffrey Dean. Efficient estimation of word representations in vector space. *CoRR*, abs/1301.3781, 2013.

[16] PedregosaF., VaroquauxG., GramfortA., MichelV., ThirionB., GriselO., BlondelM., PrettenhoferP., WeissR., DubourgV., VanderplasJ., PassosA., CournapeauD., BrucherM., PerrotM., and DuchesnayE. Scikit-learn: Machine learning in Python. *Journal of Machine Learning Research*, 12:2825–2830, 2011.

[17] Patrick Roocks. Computing pareto frontiers and database preferences with the rpref package. *The R Journal*, 8(2):393–404, dec 2016.

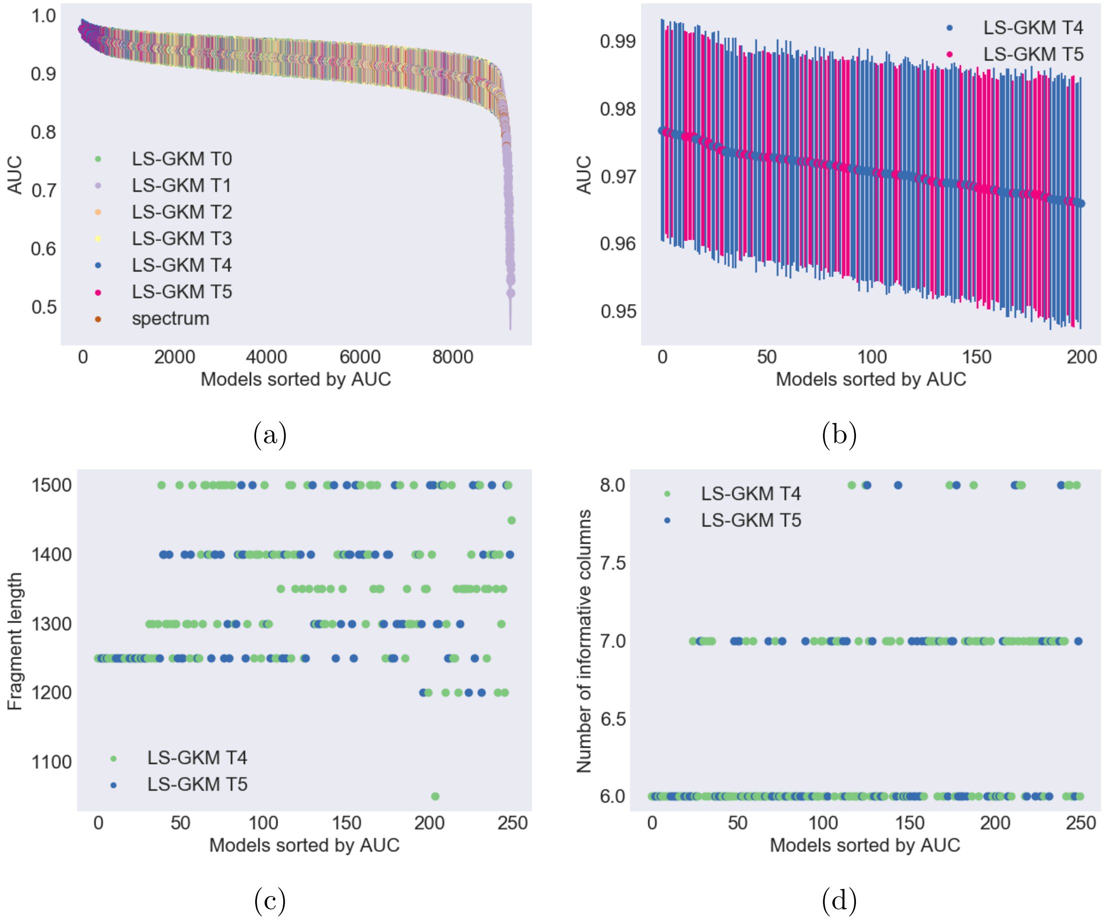

Supplementary Figure 1: **Comparison of the *oriC* classification performance of different k-mer SVMs**. **(a)** Visualization of the performance of all models. Error bars represent confidence intervals calculated using a permutation test. **(b)** Visualization of the performance of the 200 best models as measured by AUC. Error bars represent confidence intervals calculated using a permutation test. **(c)** Visualization of the fragment length the 250 best models (as measured by AUC) were trained on. **(c)** Visualization of values of the number of informative columns that was chosen as parameter for the 250 best models (as measured by AUC).

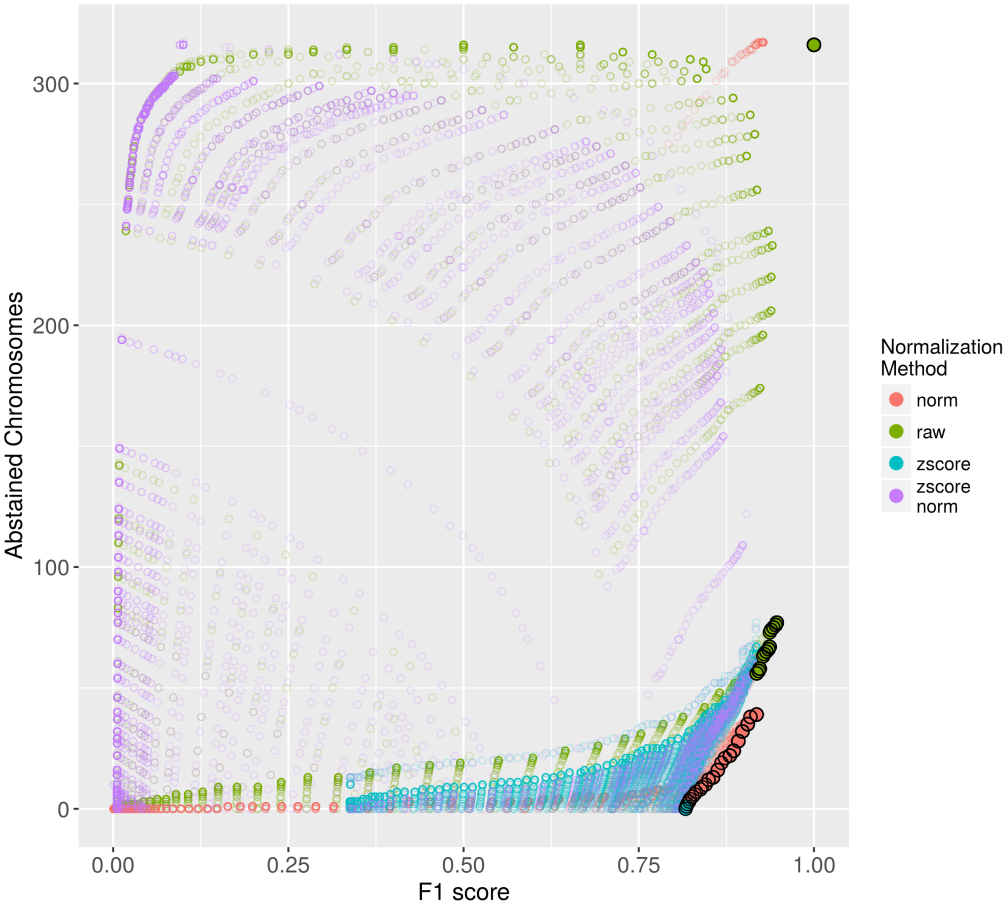

Supplementary Figure 2: **Relationship between F1 score and abstaining space given different normalization methods on the training dataset.** The number of rejected chromosomes is the number of chromosomes, for which the positive fragment is scored between the two cutoffs and is thus abstained. Every dot represents one set of cutoff parameters and a normalization method; dots with black border represent parameter sets on the Pareto front.

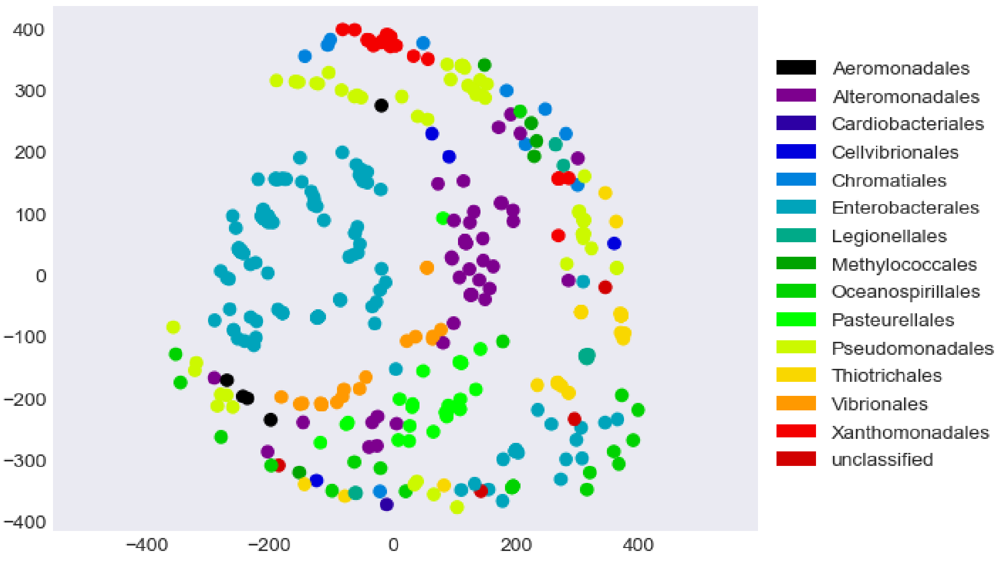

(a)

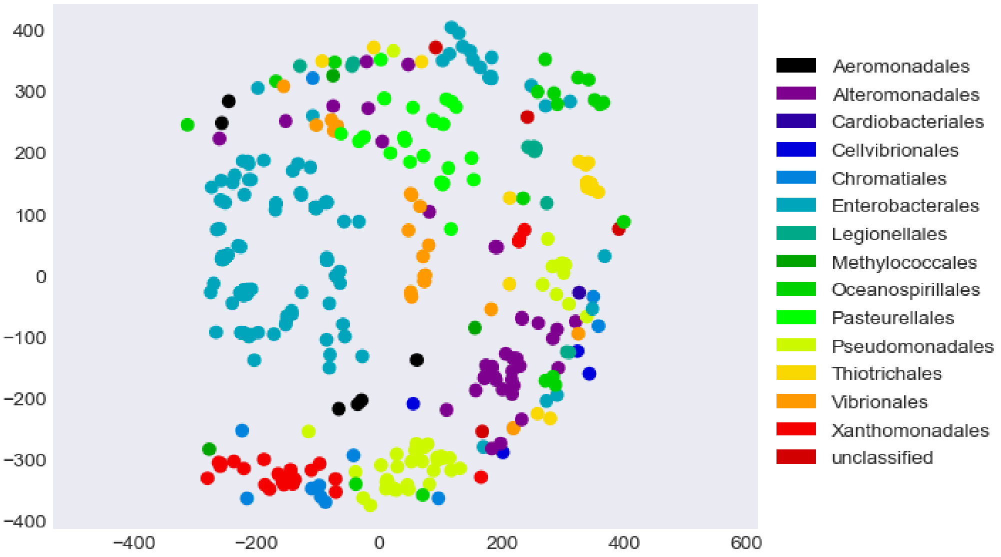

(b)

Supplementary Figure 3: **Consistency of the sequences present in *oriC* databases**. Multidimensional scaling representation of the distance matrix calculated from all-vs-all pairwise sequence alignments of sequences from *oriC* databases; **(a)** gammaBOriS (used on chromosomes that DoriC is created from) and **(b)** DoriC.

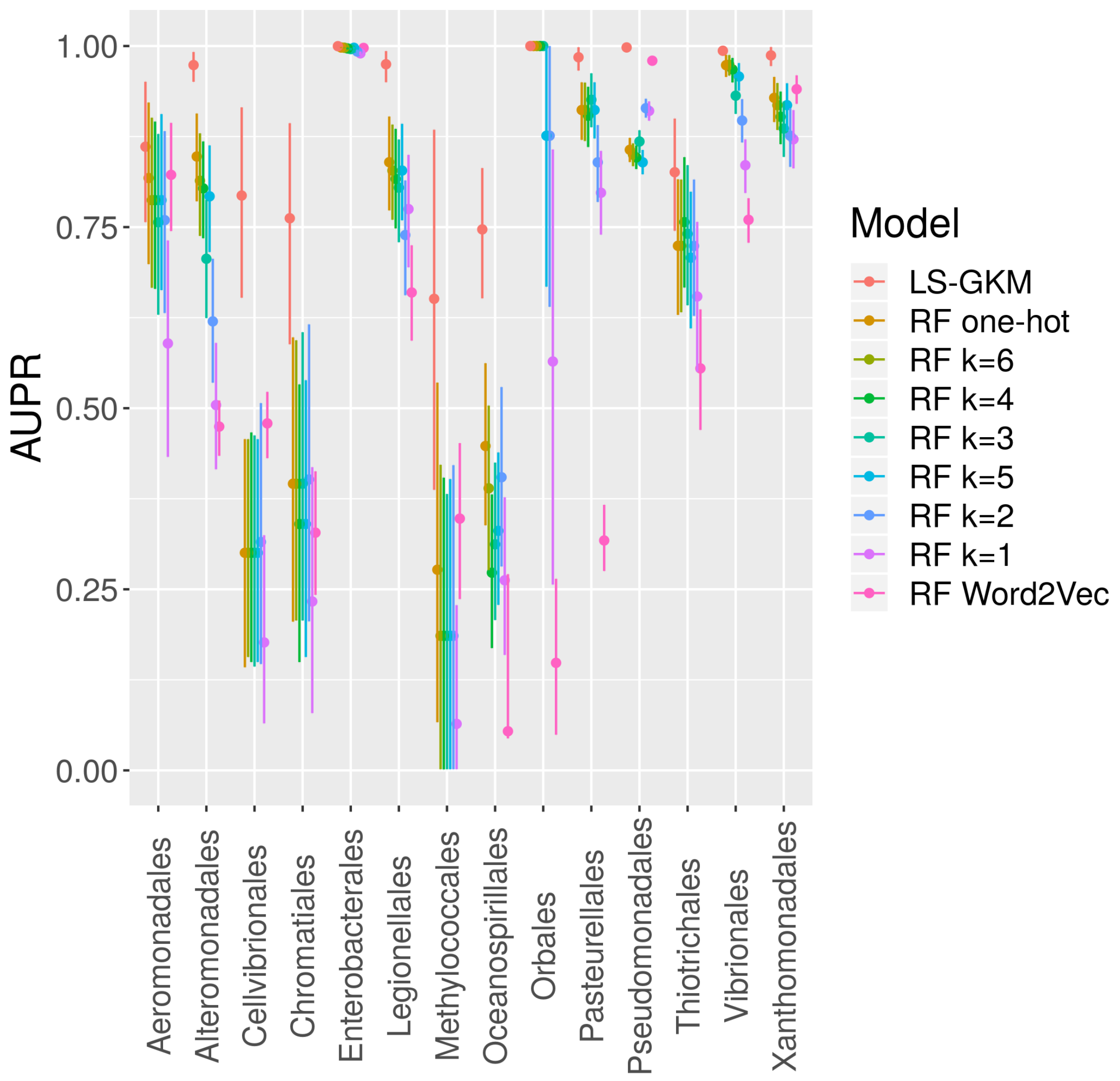

Supplementary Figure 5: **Comparison of *oriC* taxonomic classification performance of different models for gammaproteobacterial orders**. Area under the precision-recall curve (AUPR) values were calculated from predictions of the test dataset.

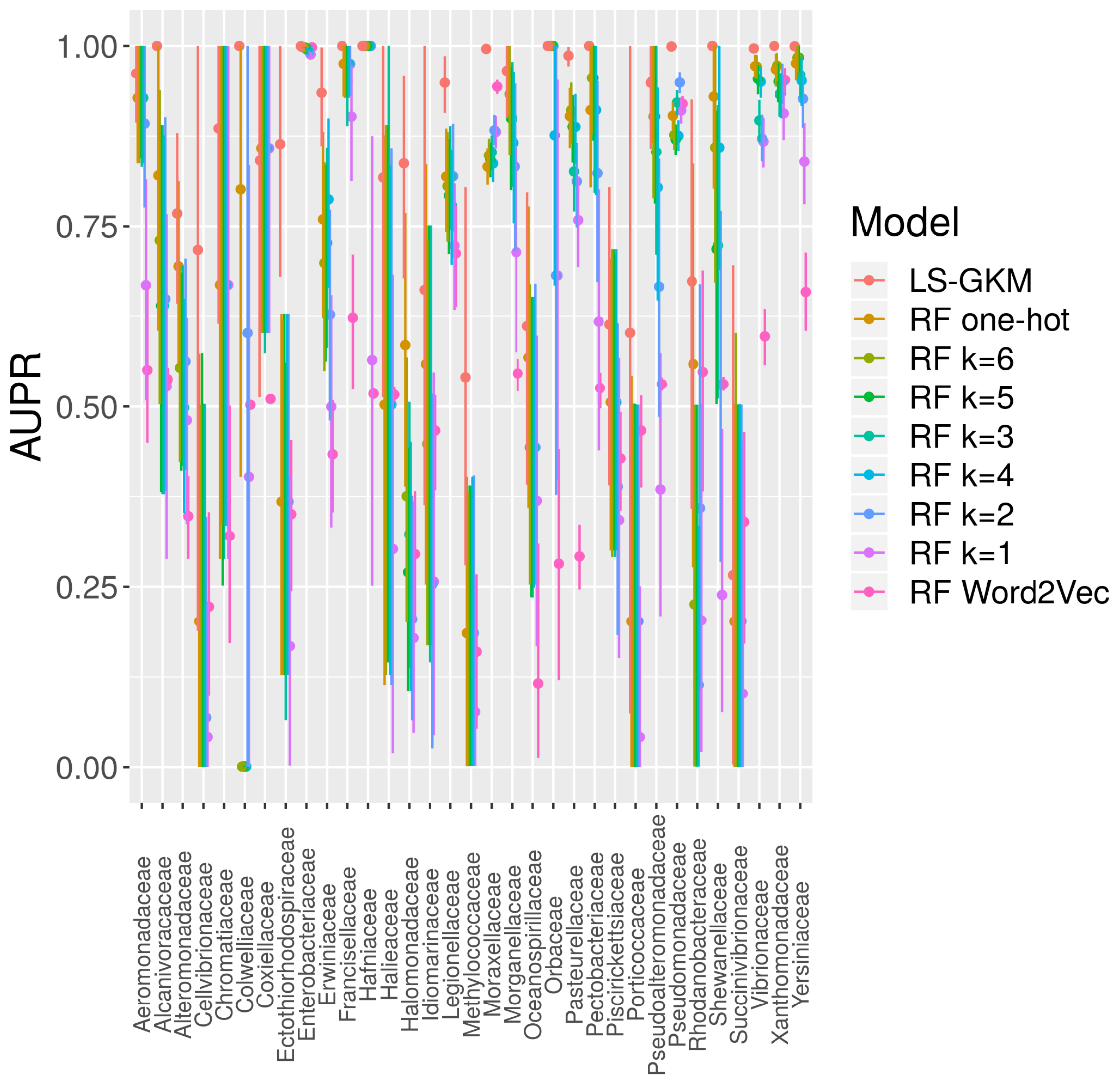

Supplementary Figure 6: **Comparison of *oriC* taxonomic classification performance of different models for gammaproteobacterial families**. Area under the precision-recall curve (AUPR) values were calculated from predictions of the test dataset.

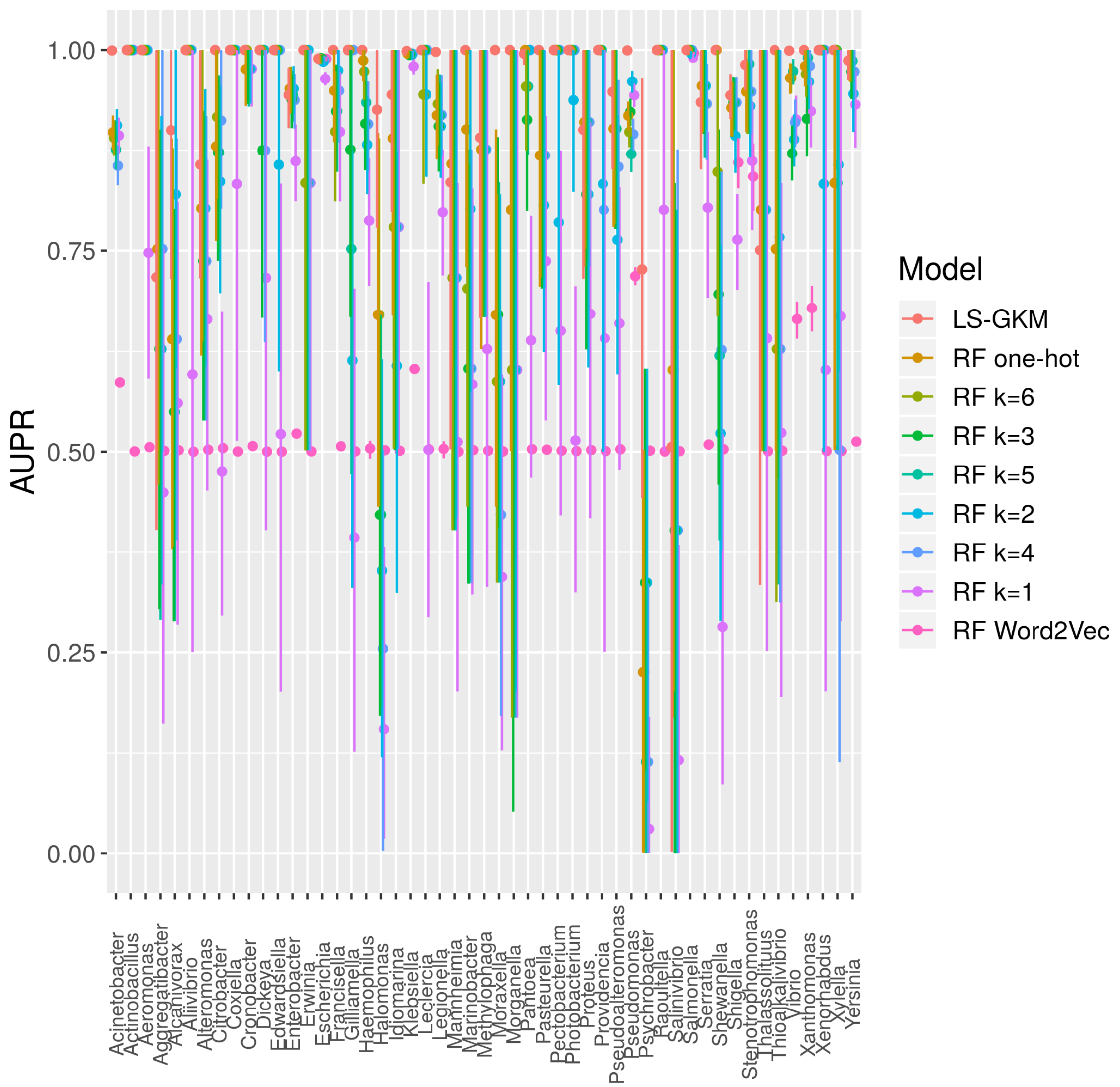

Supplementary Figure 7: **Comparison of *oriC* taxonomic classification performance of different models for gammaproteobacterial genera**. Area under the precision-recall curve (AUPR) values were calculated from predictions of the test dataset.

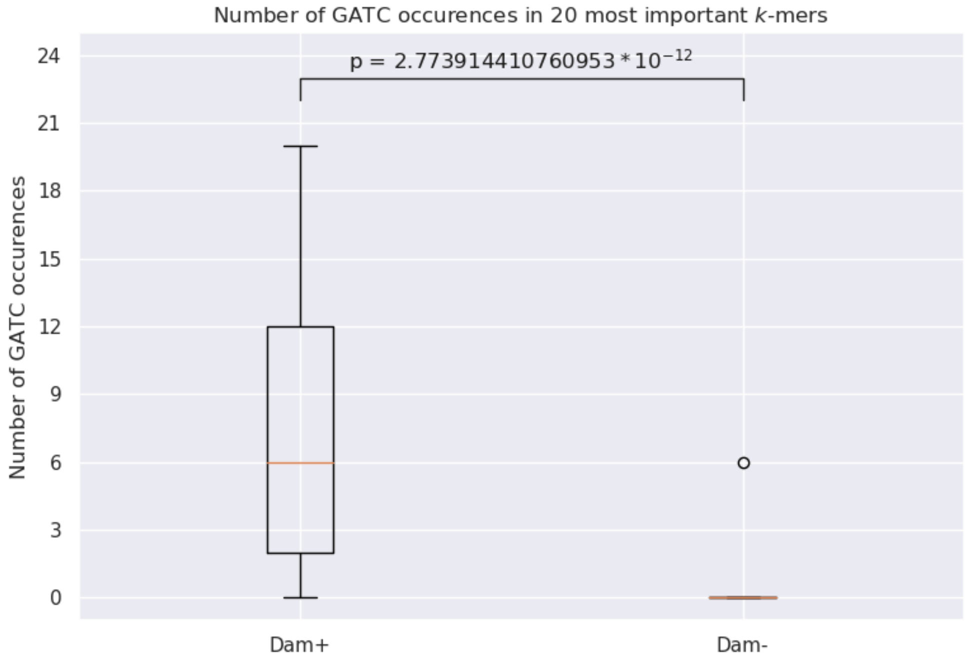

Supplementary Figure 8: **Number of GATC occurrences in the 20 most important *k*-mers for organisms in and outside of the "Dam clade".**. Taxonomic groups containing and not containing a Dam homologue were chosen as described in Brezellec *et al.*, 2006 [3]: Dam+ is composed of Vibrionales, Enterobacterales, Pasteurellales, while Dam- is composed of Pseudomonadales, Legionellales, Xanthomonadales.
